# Comprehensive Screening of Salinomycin in Feed and Its Residues in Poultry Tissues Using Microbial Inhibition Tests Coupled to Enzyme-Linked Immunosorbent Assay (ELISA)

**DOI:** 10.3390/foods13111661

**Published:** 2024-05-25

**Authors:** Daniela Spišáková, Ivona Kožárová, Simona Hriciková, Slavomír Marcinčák

**Affiliations:** Department of Food Hygiene, Technology and Safety, University of Veterinary Medicine and Pharmacy in Košice, Komenského 73, 04181 Košice, Slovakia; daniela.spisakova@student.uvlf.sk (D.S.); simona.hricikova@student.uvlf.sk (S.H.); slavomir.marcincak@uvlf.sk (S.M.)

**Keywords:** poultry, meat, salinomycin, residues, screening, microbial inhibition tests, ELISA

## Abstract

Salinomycin is a coccidiostat approved as a feed additive for the prevention of coccidiosis in poultry. Official control of its residues is set by the Commission Delegated Regulation (EU) 2022/1644. The aim of our study was to assess the suitability of three microbial inhibition tests (MITs), Premi^®^Test, Explorer 2.0, and the Screening Test for Antibiotic Residues (STAR) linked to the enzyme-linked immunosorbent assay (ELISA), for the screening of salinomycin residues in the tissues of broiler chickens (breast and thigh muscle, heart, liver, gizzard, kidneys, lungs, spleen, skin, and fat) fed commercially produced feed containing 70 mg.kg^−1^ of salinomycin in the complete feed. The first residue screening (Sampling A) was performed on the last day of administration of the salinomycin-medicated feed (day 30), and the second screening (Sampling B) was performed on the day of slaughter (day 37) after the expiry of the withdrawal period with the feeding of non-medicated feed. Based on the quantitative confirmation of salinomycin residues in the examined chicken tissues by the ELISA method (Sampling A from 0.025 to 0.241 mg.kg^−1^; Sampling B from 0.003 to 0.076 mg.kg^−1^), all the MITs with the preference of the bacterial strain *Bacillus stearothermophilus* var. *calidolactis* ATCC 10149 demonstrated the ability to detect the residues of salinomycin in the examined tissues of broiler chickens at the level of the maximum residue limits set from 0.015 to 0.150 mg.kg^−1^ by Commission Implementing Regulation (EU) 2017/1914 and confirmed the relevance of their sensitivity to the coccidiostat salinomycin.

## 1. Introduction

Coccidiosis is a highly contagious parasitic disease of the intestinal tract of many animal species including poultry, sheep, cattle, pigs, and rabbits. Even today, coccidiosis is one of the most serious diseases in industrial poultry farming. The protozoa responsible for coccidiosis belonging to the genus *Eimeria* and *Isospora* spread rapidly through contact with infected droppings. The disease can lead to significant economic losses in the agricultural industry, attributed to reduced animal production and increased mortality. As long as prevention is considered to be more important than treatment, the most typical and effective way to prevent coccidiosis is prophylactic medication in feed using coccidiostats as feed additives determined by Regulation (EC) No 1831/2003 on additives for use in animal nutrition [1,2,3,4].

Feed additives are substances, microorganisms, or preparations different from feed raw materials and premixes, which are intentionally added to feed or water. These substances must favorably influence the properties of feed and the properties of animal products, satisfy the nutritional needs of animals, favorably influence animal production and the efficiency of animals, and, in the case of coccidiostats, also destroy or inhibit the growth of protozoa [4]. Each coccidiostat can be used in the prescribed concentration and during a certain time interval for broilers and young chickens, but not for layers. Chickens for fattening receive coccidiostats in feed throughout their life, with the exception of the withdrawal period before slaughter [5].

Effective coccidiostats are currently indispensable to protect the health and welfare of poultry and other species against coccidiosis. Coccidiostats can be grouped into two major classes, namely polyether ionophore antibiotics and synthetic products not of an ionophoric nature, as well as combinations of different classes [6]. Salinomycin is a polyether monocarboxylic acid produced by the fermentation of the fungi *Streptomyces albus* and *Streptomyces aureofaciens*. It belongs to the group of polyether ionophore antibiotics mainly used for the prevention of coccidiosis in poultry. Salinomycin shows high antimicrobial activity against Gram-positive bacteria, *Clostridium perfringens*, mycobacteria, and some filamentous fungi [7,8]. Salinomycin is safe for broiler chickens at a dose of 70 mg.kg^−1^ complete feed and for layer chickens at a dose of 50 mg.kg^−1^ complete feed during the first 12 weeks of life. Salinomycin is absorbed and extensively metabolized, whereas metabolites have reduced ionophoric activity [9].

The widespread use of different coccidiostats results in a considerable risk of their residues remaining in edible tissues. Coccidiostats residues in food exhibit undesirable toxicological effects, such as teratogenicity, hepatotoxicity, or neurotoxicity, at high doses in laboratory animals [10,11]. Carboxylic acid ionophores are potent pharmacological agents, exerting marked cardiovascular effects in experimental animal systems. Most of these effects have been characterized using monensin as the model for the whole group. The principal effect is an increase in coronary flow, indicative of coronary dilatation. It has been estimated that a threshold dose for increased coronary flow in dogs, following injection of monensin, is 1.0 mg.kg^−1^. The threshold dose in man, following oral administration of monensin, in food, will inevitably exceed 1.0 mg.kg^−1^. In normal individuals, coronary dilatation is unlikely to have any adverse effect. However, it has been suggested that victims of coronary artery disease may be at an increased risk [12]. In order to protect public health, maximum residue limits (MRLs) for all pharmacologically active substances including coccidiostats in foodstuffs of animal origin are established in accordance with generally recognized principles of safety assessment, taking into account toxicological risks, environmental contamination, and the microbiological and pharmacological effects of residues [13,14]. The safety of pharmacologically active substances for animals, users, consumers, and the environment is assessed by the European Food Safety Authority (EFSA). Part of the safety assessment is also the setting of MRLs for coccidiostats in animal products of target and non-target animal species [15]. MRLs for coccidiostats are currently set as follows: (1) MRLs for coccidiostats as veterinary medicinal products are laid down by Regulation (EU) 37/2010, specifying the species of animal and the monitored matrix [13]; (2) MRLs for those coccidiostats that are approved as feed additives in poultry fattening are laid down by Regulation (EC) No 1831/2003 on additives for use in animal nutrition [4]; (3) since cross-contamination of non-medicated and medicated feed is not excluded, MRLs for coccidiostats in food of animal origin from animal species other than poultry are given in Commission Regulation (EC) No 124/2009 [16]. MRLs of salinomycin in the relevant foodstuffs of animal origin are specified by Commission Implementing Regulation (EU) 2017/1914 of salinomycin sodium as a feed additive for chickens for fattening and chickens reared for laying and repealing [17].

The widespread use of pharmacologically active substances in food animals emphasizes the determination of the presence of their residues in products of animal origin. The screening of residues of veterinary drugs in products of animal origin, as well as the development of new methods for their detection, is very important for the protection of human health and ensuring the quality and safety of food [5,18]. Commission Delegated Regulation (EU) 2022/1644 lays down specific requirements for the performance of official controls on the use of pharmacologically active substances authorized as veterinary medicinal products or as feed additives and of prohibited or unauthorized pharmacologically active substances and residues thereof [19]. Commission Implementing Regulation (EU) 2021/808 lays down rules concerning the methods of analysis of pharmacologically active substances used in food-producing animals and the interpretation of results, as well as the methods to be used for sampling and repealing [20]. Practical arrangements for the performance of official controls as regards the use of pharmacologically active substances authorized as veterinary medicinal products or as feed additives and of prohibited or unauthorized pharmacologically active substances and residues thereof on the specific content of multi-annual national control plans and specific arrangements for their preparation is determined by Commission Implementing Regulation (EU) 2022/1646 [21].

A successful screening strategy for the residues of pharmacologically active substances in food of animal origin consists of two steps: the initial screening and the subsequent use of the confirmatory method. The initial screening requires a simple, fast, accurate, and sensitive method for a safe and operative guarantee of food safety and human health. Initial screening methods ensure the selection of positive samples from a large number of initially tested samples [22]. Screening methods include microbial inhibition tests in tube [23,24] or plate [25] format [10,26,27,28]. These methods are based on the detection of residues of inhibitory substances in a wide range of matrices, while the principle is the inhibition of the growth of the test organism by the action of the antibiotic present in the examined sample. Since in the case of screening it is only a matter of confirming or refuting the presence of inhibitory substances in the sample and thus it is a qualitative or semi-quantitative determination, to confirm the result it is necessary to use confirmatory methods based either on the chromatographic (LC-MS/MS) [29,30,31,32,33,34,35] or immunological principles (enzyme-linked immunosorbent assay—ELISA) [10,35,36].

Coccidiostats belong to antimicrobial substances, whose residue control is legally set, but even so, no screening method is directly approved for its residue detection. This fact offers room for the development of new methods or the validation of available methods precisely for the purpose of initial screening of coccidiostat residues in products of animal origin. Microbial inhibition tests in tube or plate format are validated for the detection of ß-lactam residues, cephalosporin, macrolide, tetracycline, sulphonamide, aminoglycoside, quinolone, amphenicol, and polypeptide antibiotics in food and feed in line with EU MRLs [37]. Polyether ionophores show antimicrobial effectiveness, and since they are the only antimicrobial substances that have maintained approval for use in poultry nutrition after the ban on the use of antibiotics as feed additives since 1 January 2006, the presence of coccidiostat residues must be realistically considered when examining poultry meat for antibiotic residues in the first stage of residue screening with microbial inhibition tests.

Because the use of coccidiostats in poultry nutrition will not be phased out in the near future and only confirmatory analysis is still used for the control of coccidiostats in poultry meat, the aim of this study was to screen salinomycin residues in the tissues of broiler chicken by three in-practice, approved microbial inhibition tests, Premi^®^Test (R–Biopharm AG, Darmstadt, Germany), Explorer 2.0 test (Zeulab S.L., Zaragoza, Spain), and Screening Test for Antibiotic Residues (STAR) [25,38], and based on the quantitative confirmation of the results obtained by a competitive enzyme immunoassay Ionophore EIA (EuroProxima B.V., Arnhem, The Netherlands) and confirmation of the sensitivity of the respective microbial inhibition test, contribute to the creation of a comprehensive and reliable two-stage system in coccidiostat residue analysis.

## 2. Materials and Methods

### 2.1. Sample Material

Samples of commercial feed mixtures (CFM) BR2 (containing the coccidiostat salinomycin at the concentration of 70 mg.kg^−1^ in complete feed) and BR3 (without coccidiostat) (De Heus a.s, Bučovice, Czech Republic) and samples of the tissues of broiler chickens (breast muscle, upper and lower thigh muscle, heart, liver, gizzard, kidneys, lungs, spleen, skin, and fat) for analysis were provided to us from an experiment (approval protocol No EKVP/2022-08) carried out as part of project No. APVV-18-0039 at our workplace. Tissue samples were obtained from a control group of broiler chickens (hybrid ROSS), which, as part of this experiment, were fed the standard above-mentioned feed mixtures without the addition of any other substance, except for salinomycin, affecting the production indicators of poultry during fattening. The samples were taken from three broiler chickens killed by manual cervical dislocation followed by bleeding on the 30th day of fattening—Sampling A (the last day of feeding with CFM BR2 containing coccidiostat salinomycin)—and on the 37th day of fattening—Sampling B (the day of slaughter after the expiry of the withdrawal period during which the feed mixture BR3 without coccidiostat was administered). The obtained tissues were packed, labeled, and stored at −20 °C until analysis.

### 2.2. Methods

Microbial inhibition tests: Premi^®^Test (R–Biopharm AG, Darmstadt, Germany) [38]; Explorer 2.0 test (Zeulab S.L., Zaragoza, Spain); Screening Test for Antibiotic Residues (STAR) [38].

Enzyme-linked immunosorbent assay (ELISA): Ionophore EIA (EuroProxima B.V., Arnhem, The Netherlands).

### 2.3. Procedure of Tube Tests Premi^®^Test and Explorer 2.0

#### 2.3.1. Principle

The Premi^®^Test and Explorer 2.0 tests are based on the inhibition of microbial growth. The kits are supplied in a single-tube format. Each tube contains an agar medium spread with *Geobacillus thermophile* spores and a pH indicator. When the test is incubated at 65 °C, spores germinate and cells grow, producing acid and changing the agar pH. Variations in pH will produce changes in the agar color from purple to yellow. When samples contain inhibitors at higher concentrations than the limit of detection, the bacteria will not grow and color changes will not be observed.

#### 2.3.2. Preparation of the Samples

The tissue sample (3 g) was dipped into a plastic tube, placed into a beaker with water, and heated in a microwave oven set to “Defrosting” for 3 ± 1 min. The meat juice obtained was collected into a new tube and used in the following process.

The feed sample (10 g) was ground and 30 mL of sterile demineralized water was added. The mixture was mixed for 30 min at 20–25 °C by shaking and consequently centrifuged for 10 min at 3000× *g*. The supernatant was transferred into a new tube and used for further analysis.

#### 2.3.3. Screening of the Samples

Premi^®^Test and Explorer 2.0 procedures were performed in accordance with the manufacturer’s instructions. The required number of ampoules was separated using scissors, labeled, and the foil was removed. A total of 100 µL of the sample was pipetted onto the agar in the ampoule and allowed to stand at room temperature for 20 min for pre-diffusion. In the case of kidney, liver, and feed samples, Premi^®^Test ampoules were pre-incubated at 80 °C for 10 min. After pre-incubation, the sample was rinsed from the ampoule twice with demineralized water and the ampoules were sealed with adhesive film to prevent evaporation. Premi^®^Test ampoules were incubated in a digital dry bath (Labnet Accublock Digital Dry Bath D 1200, Labnet, Edison, NJ, USA) at 65 °C ± 0.5 °C for approximately 3 to 3.5 h, until the negative control changed from purple to yellow. Explorer 2.0 ampoules were incubated at 65 °C ± 1 °C using the e-Reader (Zeulab S.L., Zaragoza, Spain) by selecting the automatic assay linked to the Explorer 2.0 procedure with the use of a negative control.

#### 2.3.4. Reading the Results of the Premi^®^Test

The procedure was terminated when the negative control sample changed the color of the agar from purple to yellow. A color change from purple to yellow indicated that the sample did not contain any antibiotic residues or that the residue concentration was below the detection limit of the respective test. If the color remained purple, the sample contained antibiotic residues at a concentration above the detection limit of the respective test. The test results were evaluated eye-metrically using the color card supplied by the manufacturer as part of the kit.

#### 2.3.5. Reading the Results of the Explorer 2.0 Test

The procedure was terminated automatically, and the results were read and displayed using an e-Reader. The e-Reader device detects the color change in the negative control by photometric (595 and 650 nm) reading, determines the cut-off value, and automatically terminates the incubation time of the samples with a positive sample expressed as a value ≥56 and a negative sample as a value <56. A change in color from purple to yellow indicated that the sample did not contain any antibiotic residues or that the residue concentration was below the detection limit of the respective test. If the sample contained antibiotic residues in a concentration at or above the detection limit of the test, the color remained purple.

### 2.4. Procedure of Plate Test STAR

#### 2.4.1. Principle

The STAR method is based on inoculating five bacterial microorganisms sensitive to antibacterial substances into the agar medium in Petri dishes. The preferential sensitivity of the bacterial microorganisms used to selected antibacterial substances is as follows: *Escherichia coli* ATCC 11303 to quinolones, *Bacillus cereus* ATCC 11778 to tetracyclines, *Bacillus subtilis* BGA to aminoglycosides, *Bacillus stearothermophilus* var. *calidolactis* ATCC 10149 to beta-lactams and sulfonamides, and *Kocuria rhizophila* ATCC 9341 to macrolides and beta-lactams. Paper discs containing an antibacterial substance as a control test and a slice of tissue sample were placed onto the surface of the inoculated medium and then incubated at the optimal temperature for the growth of the test organism. After diffusion, the present antibacterial substance may produce an inhibition zone around the sample by inhibiting the growth of the test organism.

#### 2.4.2. Preparation of Test Plates

The STAR procedure was completed according to STAR protocol by the Community Reference Laboratory AFFSA in Fougeres (France) [25]. This method is also approved as an official method of laboratory diagnostics of The State Veterinary and Food Administration of the Slovak Republic [38].

Four different agar media were inoculated with five different bacterial strains as follows: (1) test agar pH 8.0 (Merck 10664; Merck, Darmstadt, Germany) was inoculated with *Escherichia coli* ATCC 11303 (Czech Collection of Microorganisms, Brno, Czech Republic) and *Kocuria rhizophila* ATCC 9341 (Czech Collection of Microorganisms) individually; (2) test agar pH 6.0 (Merck 10663) was inoculated with *Bacillus cereus* ATCC 11778 (Czech Collection of Microorganisms); (3) Antibiotic medium 11 (Difco 259310; Difco, Detroit, MI, USA) was inoculated with *Bacillus subtilis* BGA (Merck 10649); and (4) DST test agar pH 7.4 (CM 261; Oxoid, Basingstoke, UK) was inoculated with *Bacillus stearothermophilus* var. *calidolactis* ATCC 10149 (Merck 1.11499). After the preparation of bacterial suspensions and media according to the manufacturer’s instructions, the inoculated media were added to Petri dishes in a volume of 5 mL, individually. The agar was allowed to solidify, and the plates thus prepared were used for further residue analysis.

#### 2.4.3. Preparation and Screening of the Samples

A cylindrical core with a diameter of 8 mm and a length of approximately 2 cm was obtained from each frozen tissue sample using a sterile cork borer (Ø 9 mm) and cut into 2 mm thick slices with a sterile scalpel. The cut slices were placed in duplicates facing each other on the surface of the test agar plates. The feed samples in the amount of 30 µL were pipetted onto 9 mm paper discs in duplicates placed on the surface of the agar medium of the test plates, respectively. Plates were incubated under the conditions determined by the method: plates inoculated with *Bacillus stearothermophilus* var. *calidolactis* ATCC 10149 for 12–15 h at 55 °C, plates inoculated with *Bacillus subtilis* BGA and *Bacillus cereus* ATCC 11778 for 18 h at 30 °C, plates inoculated with *Escherichia coli* ATCC 11303 for 18 h at 37 °C, and plates inoculated with *Kocuria rhizophila* ATCC 9341 for 24 h at 37 °C.

#### 2.4.4. Reading the Results

The sample was considered positive if the inhibition zone created around the sample on agar test plate was equal to or greater than 4 mm on plate inoculated with *Bacillus stearothermophilus* var. *calidolactis* ATCC 10149 and/or equal to or greater than 2 mm on plates inoculated with *Bacillus subtilis* BGA, *Bacillus cereus* ATCC 11778, *Escherichia coli* ATCC 11303, and *Kocuria rhizophila* ATCC 9341. The inhibition zone width was measured as the distance between the edge of the tissue section or disc and the outer border of the inhibition zone in mm using a digital caliper (Mitutoyo, Kawasaki, Japan) with an accuracy of 0.01 mm.

#### 2.4.5. Verification of the Sensitivity of Bacterial Strains to Control Antibiotic Solutions

The specific positive control of the antibiotic solution was used for each test bacteria as follows: (1) ciprofloxacin in a concentration of 100 µg·L^−1^ for *Escherichia coli* ATCC 11303 with a presumed inhibition zone of 5.5 ± 1.5 mm, (2) chlortetracycline in a concentration of 200 µg·L^−1^ for *Bacillus cereus* ATCC 11778 with a presumed inhibition zone of 6.0 ± 1.5 mm, (3) streptomycin in a concentration of 2000 µg·L^−1^ for *Bacillus subtilis* BGA with a presumed inhibition zone of 4.5 ± 1.5 mm, (4) sulphamethazine in a concentration of 1000 µg·L^−1^ for *Bacillus stearothermophilus* var. *calidolactis* ATCC 10149 with a presumed inhibition zone of 5.0 ± 1.5 mm, and (5) tylosin in a concentration of 1000 µg·L^−1^ for *Kocuria rhizophila* ATCC 9341 with a presumed inhibition zone of 5.5 ± 1.5 mm. Antibiotic solutions in the amount of 30 µL were pipetted onto 9 mm paper discs in duplicates placed on the surface of the agar medium of the test plates. Plates were incubated as follows: *Bacillus stearothermophilus* var. *calidolactis* ATCC 10149 plate for 12–15 h at 55 °C, *Bacillus subtilis* BGA and *Bacillus cereus* ATCC 11778 plates for 18 h at 30 °C, *Escherichia coli* ATCC 11303 plate for 18 h at 37 °C, and *Kocuria rhizophila* ATCC 9341 plate for 24 h at 37 °C.

#### 2.4.6. Determining the Sensitivity of the Used Methods to Salinomycin

Salinomycin standard solutions were prepared to the concentrations of 500, 100, 75, and 50 µg·L^−1^ by dissolving salinomycin standard (Sigma-Aldrich S4526 PtyLtd., Darmstadt, Germany) in sterile demineralized water. The sensitivity of Premi^®^Test, Explorer 2.0, and the STAR method to salinomycin were tested using procedures for individual methods described above.

### 2.5. ELISA

#### 2.5.1. Principle

The microtiter plate-based ELISA kit consists of 12 strips, each containing 8 wells, precoated with rabbit antibodies to sheep IgG. Antibodies (sheep polyclonal anti-salinomycin antibodies), horseradish peroxidase-labeled salinomycin (enzyme-conjugated salinomycin-HRP), and salinomycin standard solutions or samples are added to the precoated wells, followed by a single incubation step. The antibodies are bound by the immobilized rabbit anti-sheep antibodies, and simultaneously, the salinomycin-HRP and salinomycin present in the standard solutions or salinomycin present in the samples compete for binding to the anti-salinomycin antibody (competitive enzyme immunoassay). After incubation for one hour, non-bound reagents are removed in a washing step. The amount of bound salinomycin-HRP is visualized by the addition of the enzyme substrate/chromogen (peroxide/tetramethylbenzidine, TMB). During the incubation, the colorless chromogen is converted by the enzyme into a blue reaction product. This blue color is inversely proportional to the amount of bound salinomycin. The more salinomycin present in the standard solution or sample, the less color is developed. The color development is stopped by the addition of sulphuric acid. In an acidic environment, the blue color changes into yellow. The color intensity is measured photometrically at 450 nm.

#### 2.5.2. Preparation of the Samples

Approximately 50 g of the sample was ground and pulverized into a fine homogenous mixture. Precisely 1 g of homogenized tissue sample was weighed into a tube and 5 mL of 100% methanol (67561 Merck, Darmstadt, Germany) was added. The mixture was shaken for 15 min and centrifuged for 10 min at 2000× *g*. A 1 mL methanol layer was pipetted into a glass tube and evaporated under a mild stream of nitrogen at 50 °C using water-bath nitrogen-blowing (Turbo-Vap LV, Zymarck, Germany). The residue was dissolved in 200 μL of 100% methanol and vortexed for 15 s. Subsequently, 800 μL of PBS was added and the mixture was vortexed for 10 s and centrifuged for 1 min 2000× *g*.

#### 2.5.3. Screening of the Samples

The salinomycin standard solutions in the concentrations of 1.25, 2.5, 5, 10, 20, and 40 ng.mL^−1^ in an amount of 50 μL were pipetted in duplicates into the wells of the microtiter plate, respectively. In a similar manner, 50 μL of each prepared sample solution was pipetted in duplicate into different wells of the microtiter plate. The conjugate (salinomycin-HRP) in the amount of 25 μL was added to all wells. Consequently, 25 μL of the antibody solution was added to all wells, the microtiter plate was sealed, and the plate was shaken for a few seconds on a microtiter plate shaker. After, the plate was incubated for 1 h in the dark at 4 °C (2–8 °C). After incubation, the solution from the microtiter plate was discarded and wells were washed 3 times with rinsing buffer. A 100 μL of substrate/chromogen solution was added to all wells and the plate was incubated for 30 min at room temperature (approximately 20–25 °C). Immediately after adding 100 μL of the stop solution to each well, the values of the absorbance were read at 450 nm using an ELISA microplate reader (Labtech LT-4000, Schönwalde-Glien, Germany).

### 2.6. Data Analysis

Data analysis of the STAR method was performed using Microsoft Office Excel 2019. The values of inhibition zones were expressed as the mean ± standard deviation (SD) of six measures.

Data analysis of the quantitative ELISA method was performed automatically with the LabTech Manta LML software (Schönwalde-Glien, Germany). The final results were obtained by recalculating the readings with respect to the respective dilution used. The results were expressed in mg.kg^−1^ of the relevant tissue.

## 3. Results

The results of screening salinomycin in commercial feed mixtures and its residues in the examined tissues of broiler chickens are presented in Table 1, Table 2, Table 3, Table 4 and Table 5.

### 3.1. Premi^®^Test, Explorer 2.0 Test

The results obtained by determining the presence of salinomycin and its residues using the commercially available Premi^®^Test and Explorer 2.0 test are presented in Table 1. Using visual reading of the Premi^®^Test results and automatic reading of the results of the Explorer 2.0 test using an electronic device e-Reader, all chicken tissue samples in Sampling A were evaluated as positive for antibiotic residues. In Sampling B, all chicken tissue samples were evaluated as negative for antibiotic residues. Feed sample BR2 was evaluated as positive, and = feed sample BR3 was evaluated as negative. The results of both commercially available tube tests were identical.

### 3.2. STAR

Table 2 presents the results obtained by the STAR method. By examining tissue samples in Sampling A, the presence of inhibition zones was detected in all samples *on Bacillus stearothermophilus* var. *calidolactis* ATCC10149 and *Kocuria rhizophila* ATCC 9341 plates and in liver and lungs samples on *Bacillus subtilis* BGA plates. No inhibition zones were detected on *Bacillus cereus* ATCC 11778 and *Escherichia coli* ATCC 11303 plates. The samples on the plates with the bacterial strain *Bacillus stearothermophilus* var. *calidolactis* ATCC 10149 were considered positive if they showed an inhibition zone equal to or greater than 4 mm, and on plates with the bacterial strain *Kocuria rhizophila* ATCC 9341, *Bacillus subtilis* BGA, *Bacilus cereus* ATCC 11778, and *Escherichia coli* ATCC 11303, an inhibition zone equal to or greater than 2 mm. Based on these criteria, all tissue samples were evaluated as positive on *Bacillus stearothermophilus* var. *calidolactis* ATCC 10149 plates; liver and lung samples were evaluated as positive on *Bacillus subtilis* BGA plates; and breast muscle, upper and lower thigh muscle, liver, kidney, and spleen samples were evaluated as positive on *Kocuria rhizophila* ATCC 9341. Other tissue samples were evaluated as negative.

By examining chicken tissue samples in Sampling B, the presence of inhibition zones was detected in all samples on *Bacillus stearothermophilus* var. *calidolactis* ATCC 10149 plates; and in breast muscle, heart, liver, kidney, lungs, spleen, and skin samples on *Kocuria rhizophila* ATCC 9341 plates. Based on the criteria set for the evaluation of positivity of the STAR method, all samples were evaluated as negative.

By examining feed samples, the presence of inhibition zones was detected in the BR2 sample on *Bacillus stearothermophilus* var. *calidolactis* ATCC 10149 and *Bacillus cereus* ATCC 11778 plates. The presence of salinomycin in this commercial feed mixture was confirmed.

Table 3 declares the confirmation of the sensitivity of bacterial strains of the STAR method to the control solutions of antibiotics. The inhibition zones created around the paper discs with control antibiotic solutions were in compliance with the inhibition zones set by the method.

Table 4 shows the sensitivity of the STAR method, Premi^®^Test, and Explorer 2.0 test to salinomycin. Sensitivity to salinomycin was demonstrated by inhibiting the growth of the test bacterial strain *Bacillus stearothermophilus* var. *calidolactis* in all three methods at the lowest concentration of 50 µg·L^−1^. Salinomycin also inhibited the growth of *Bacillus cereus* ATCC 11778 at the highest tested concentration (500 µg·L^−1^).

The sensitivity of the test strain *Bacillus stearothermophillus* var. *calidolactis* to salinomycin was confirmed in tube tests (Premi^®^Test and Explorer 2.0) as well as in the case of the STAR method. When examining the samples by the STAR method, two test strains, *Bacillus stearothermophillus* var. *calidolactis* and *Kocuria rhizophila*, were mainly inhibited, while the largest zones of inhibition were formed on *Bacillus stearothermophillus* var. *calidolactis* plates. Therefore, we can consider *Bacillus stearothermophillus* var. *calidolactis* as a highly sensitive and preferential test strain for the screening of residues of pharmacologically active substances, especially coccidiostats.

### 3.3. ELISA

The presence of salinomycin residues in examined tissue samples was verified by the ELISA method (Table 5). By examining tissue samples in Sampling A, the presence of salinomycin residues was detected by the ELISA method in all investigated matrices, but positive results, i.e., above the level of MRL were detected and gradually declined in liver, spleen, lung, kidney, gizzard, heart, thigh muscle, and breast muscle samples. By examining tissue samples in Sampling B, the presence of salinomycin residues was also detected in all examined matrices; however, only lungs and spleen samples contained the salinomycin residues above the MRL. Other samples were negative for salinomycin residues. The MRL of salinomycin residues is specified by Commission Implementing Regulation (EU) 2017/1914 at the following concentrations: 0.015 mg.kg^−1^ for muscle, 0.150 mg.kg^−1^ for liver, 0.040 mg.kg^−1^ for kidney, and 0.150 mg.kg^−1^ for skin/fat [17].

## 4. Discussion

The high percentage of antibiotic use in livestock farming and the presence of drug residues in food represent a serious problem because of their harmful effects on human health [10]. In order to avoid the presence of antibiotic residues in food, they must be strictly regulated in terms of their dose or withdrawal period. “Withdrawal period means the time that must elapse between the last administration of a veterinary medicine and the slaughter or production of food from that animal, to ensure that the food does not contain levels of the medicine that exceed the maximum residue limit” [39].

A wide range of methods for the analysis of antibiotic residues in meat are currently available. In Europe, methods for official control are classified as screening and confirmatory methods. The most common surveillance programs for antibiotic residue control start by screening a large number of samples in a short time with easy and inexpensive methods. Screening methods must detect a broad spectrum of antimicrobials at the regulatory levels; ideally, no more than 5% false compliant results should be accepted. Moreover, presumptive non-compliant results must be confirmed with a suitable validated method [40].

Currently, many screening methods are available, which differ not only in the type of test strain used and the time required for determination but also in sensitivity to individual substances or groups of antibiotics [41]. An important advantage of using microbial screening methods is that they are able to detect any metabolite that exhibits antibacterial activity and represent a cost-effective and time-effective way to reduce the number of samples that need to be analyzed by expensive physicochemical confirmation [28,42,43].

Although the STAR method, Premi^®^Test, and Explorer 2.0 test are not primarily intended for the determination of salinomycin in feed and its residues in food of animal origin, all these methods developed for the screening of antibiotic residues in live animals and animal products confirmed sensitivity to salinomycin standard and proved the suitability for the screening of salinomycin residues in the tissues of broiler chickens. The test strain *Bacillus stearothermophilus* var. *calidolactis* ATCC 10149 showed sensitivity to salinomycin at a concentration of 50 µg.L^−1^ by forming an inhibition zone of 1.91 ± 0.49 mm in the case of the STAR method and by purple color in the case of the Premi^®^Test and Explorer 2.0. Similar results were demonstrated in the study published by Hricikova et al. (2024) using the Explorer 2.0 test and in the study published by Kožárová et al. (2020) using the Premi^®^Test and the STAR method. Both studies proved the suitability of *Bacillus stearothermophilus* var. *calidolactis* ATCC 10149 for the screening of salinomycin residues in tissue samples [28,43].

Visual evaluation of the results can lead to misinterpretation and possible discrepancies between the laboratory results and the results obtained by the users. Therefore, Mata et al. (2014) proposed test automation by linking a microbial assay (Explorer^®^) to an automated device (e-Reader^®^) developed for the detection of antimicrobials in tissues. The use of the device was later extended to the detection of antimicrobial substances in milk and eggs [23]. In the study of Hriciková et al. (2024), an e-Reader was used to determine screening for the presence of salinomycin residues in muscle, liver, and kidney [43].

The e-Reader represents a significant shift toward the evaluation of results as it gives the exact numerical result of the samples. The evaluation process is automated. Using spectrophotometric measurement, it determines the color change in the negative control and sets the cut-off value (“Cut-off” value—the sample contains the analyte at or above the specified target concentration/MRL/) and automatically terminates the incubation of the samples. It eliminates visual evaluation errors, saves time, and reduces false positive results.

The results obtained by MIT are confirmed in practice by confirmatory methods. ELISA was and still is one of the methods for detecting coccidiostats. Several authors have worked on the development of ELISA methods for the determination of coccidiostat residues in animal products. Shen et al. (2001) describe an ELISA assay used for the screening of maduramycin in chicken tissues such as muscle, liver, and fat in combination with an immunoaffinity column pre-purification procedure. Its sensitivity and specificity are enhanced due to the hexagonal bridging between maduramycin and the binding protein, whereby the antibody is completely displaced by maduramycin from maduramycin C6-ovalbumin. The limits of detection were 1.0 ng.g^−1^ in muscle, 2.8 ng.g^−1^ in liver, and 1.5 ng.g^−1^ in fat [44].

Another ELISA for the determination of maduramycin in chicken tissues was optimized by Shen et al. (2009) using a specific monoclonal antibody with high anti-interference ability and negligible cross-reactivity with other usually used polyether antibiotics. The detection limits were 6.5 µg.kg^−1^ in muscle and 9.2 µg.kg^−1^ in liver, respectively [45].

In 2002, Beier et al. evaluated a competitive ELISA (cELISA) method for halofuginone by comparing it with high-performance liquid chromatography (HPLC) in chicken roast samples obtained from commercial slaughterhouses. The results clearly demonstrated that the cELISA method gives comparable results to the regulatory HPLC method and that the cELISA is a useful analytical method for the analysis of halofuginone in chicken samples with a detection limit of 50 μg.kg^−1^. Out of the 473 samples analyzed, the HPLC method determined 468 samples to have less than 50 μg.kg^−1^, and the cELISA method determined 423 samples to have less than 50 μg.kg^−1^. The HPLC method determined two samples and the cELISA method determined six samples in the range of 100–160 μg.kg^−1^. Neither method determined a violative level (≥160 μg.kg^−1^) in any sample. The greatly simplified sample preparation and increased sample throughput would make the cELISA method a good screening tool for the analysis of halofuginone in chicken liver tissues [46].

Huet et al. (2005) were involved in the development of ELISA for two substances, halofuginone and nicarbazine in chicken eggs and muscle. The detection ability of the test was determined to be below 0.5 μg.kg^−1^ in eggs and 1 μg.kg^−1^ in muscle for halofuginone and below 3 μg.kg^−1^ in eggs and 10 μg.kg^−1^ in muscle for nicarbazine. Both tests were fully validated and compared with a routine LC-MS/MS method [47].

Gaudin and Laurentie (2009) evaluated the sensitivity of ELISA for nicarbazine with an evaluation of the overall measurement error. The authors concluded that the method should not be used as semi-quantitative but rather as qualitative to detect the presence of nicarbazine in eggs with a detectable limit of 20 μg.kg^−1^ [48].

In 2001, Watanabe et al. described a quantitative ELISA for salinomycin in chicken plasma, liver, and muscle. Because narasin and salinomycin have very similar chemical structures, a monoclonal antibody against salinomycin recognizes narasin with a similar affinity. The ELISA was quantified in the range of 0.05–25.6 ng.mL^−1^ and showed 50% inhibition at 0.7 ng.mL^−1^. Detection limits using the ELISA were 10 ng.g^−1^ for plasma, liver, and chicken muscle. The detection limit using the kit was 50 ng.g^−1^ for plasma and 300 ng.g^−1^ for chicken muscle [49].

In the study of Tian et al. (2017), an efficient, simple, and inexpensive cELISA based on immunomagnetic sample cleanup was developed to screen salinomycin residues in chicken muscle and liver. After simple extraction, residues in sample extracts were specifically adsorbed and the supernatant was removed by magnetic separation. Analytes retained on the beads were then released by elution prior to ciELISA. The limit of detection for salinomycin in chicken muscle and liver was 22 and 18 ng.mL^−1^. The inhibition efficiency and sensitivity of this method were compared with traditional hydrophilic-lipophilic balance column clean-up cELISA [50].

In the study of Hriciková et al. (2024,) the ELISA method was used to determine the concentration of salinomycin residues in broiler tissues under the influence of adding humic substances, fermented products, and their mixture to feed supplemented with the coccidiostat salinomycin. Residues of salinomycin were present in muscle tissues of chickens of all control and experimental groups in a range of 4.749–0.310 μg.kg^−1^. All samples had salinomycin concentration values below the level of MRLs established by European law [44].

The availability of rapid screening methods suitable for the simultaneous determination of several residues of coccidiostats in one procedure is an important goal for laboratories involved in the system of official controls. However, the analysis of coccidiostats is difficult because of their similar pharmacological effects, and coccidiostats differ from each other in their chemical structures and characteristics. In order for these immunochemical methods to be used in common routine analyses, regardless of the matrix under investigation, they must be validated using appropriate test matrices and have the required detection limit for residue screening purposes. For implementation in practice, they should also be fast, cost-effective, and reliable. They should also be able to determine several residues of coccidiostats in any food matrix at the same time and at the required level. However, it is quite difficult to combine all required properties in one test in accordance with legislative requirements [2]. Despite the above, salinomycin belongs to the group of polyether coccidiostats, which shows antimicrobial activity similar to antibiotics, and the screening of their residues can be reliably performed using MIT, which was confirmed using the ELISA test in our study.

The presence of unauthorized substances, residues of veterinary medicinal products, or chemical contaminants is mandatorily controlled. The European Food Safety Authority evaluates the monitoring of results from the screening of residues of veterinary drugs and other substances in live animals and animal products in the European Union, Iceland, Norway, and the United Kingdom (Northern Ireland) every year. As presented in another EFSA report published in 2021, overall, the percentage of non-compliant samples in 2021 (0.17%) was lower compared to the previous 12 years (0.19–0.37%). The number of analyses carried out in 2021 for antimicrobials in targeted samples was 99,167 of which 139 (0.14%) were non-compliant (156 non-compliant results). Non-compliant samples for antibacterials were reported in bovines (0.19%), ovine/caprine (0.49%), swine (0.09%), poultry (0.05%), rabbits (0.39%), milk (0.07%), and eggs (0.26%). In total, 113,536 targeted samples were analyzed for substances in group B2 (other veterinary drugs) and 142 samples (0.13%) were non-compliant. Of these in Group B2, 37,896 targeted samples were analyzed for substances in subgroup B2b (anticoccidials) of which 43 samples (0.11%) were non-compliant. Non-compliant samples for anticoccidials were reported in swine (0.06%), poultry (0.07%), rabbit meat (0.87%), ovine/caprine (0.15%), and eggs (0.42%). Salinomycin was detected in poultry. Since 2009, an important decrease has been observed in the frequency of non-compliant targeted samples for anticoccidials (B2b) in poultry. This decrease is most likely the result of the awareness and the measures that followed the implementation of the Commission Directive 2009/8/EC setting up maximum levels of unavoidable carry-over of coccidiostats in non-target feed [51].

Poultry meat has become an important consumer product worldwide and has a priority position in rational nutrition. Its production belongs to the most economical and efficient branches of animal production. Compared to other types of meat, the price of poultry meat is relatively low. In 2022, the consumption of meat per capita (in carcass weight) was 71 kg, and in comparison with 2021, recorded a decrease of only 0.1 kg (0.1%). From individual types of meat, a decrease in consumption was observed in pork from 0.8 kg to 39.0 kg (2.0%). In contrast, poultry meat consumption increased from 0.7 kg to 24.5 kg (2.9%). The consumption of other types of meat has not changed in 2022. In 2021, from the available data from Central European countries, high consumption of meat per capita was seen in Austria (88.5 kg), the Czech Republic (86.0 kg), and Germany (82.1 kg). Lower levels of consumption were recorded in Poland (75.1 kg) and Hungary (71.1 kg) [52]. Even though the consumption of poultry meat in the Slovak Republic is below the European average, we can state that this consumption is sufficient, as it is higher than the recommended annual consumption of poultry meat per person (15 kg) [53]. In general, we can state that the consumption of poultry meat belongs to the phenomena of nutrition. Therefore, it is justified that the control of residues with regard to the administration of coccidiostats is important and targeted.

## 5. Conclusions

Screening of coccidiostats in feed intended for poultry and their residues in poultry tissues is very important for the protection of public health and the guarantee of food safety. All three screening methods detected positive results and indicated the presence of salinomycin and its residues in the examined chicken tissues. The result of the screening methods was finally confirmed and quantitatively evaluated by the ELISA test. The connection between screening tests and the ELISA method clearly represents an effective tool for determining the residues of coccidiostats in poultry tissues and confirming the suitability of using the STAR method and screening tests with a preference for the Explorer 2.0 test for the relevant screening of coccidiostats and their residues within the initial screening of residues of antimicrobial substances in the tissues of food animals. Microbial inhibition tests are, so far, the only choice for the initial screening of antibiotic residues and can also be used for routine screening of coccidiostats and their residues.

## Figures and Tables

**Table 1:** Results of the screening of salinomycin and its residues in matrices using the Premi^®^Test and Explorer 2.0 test.

Sampling	Matrix	Premi^®^Test	Explorer 2.0 Test
A	Breast	+	+
	Thigh upper	+	+
	Thigh lower	+	+
	Heart	+	+
	Liver	+	+
	Gizzard	+	+
	Kidney	+	+
	Lungs	+	+
	Spleen	+	+
	Skin	+	+
	Fat	+	+
B	Breast	-	-
	Thigh upper	-	-
	Thigh lower	-	-
	Heart	-	-
	Liver	-	-
	Gizzard	-	-
	Kidney	-	-
	Lungs	-	-
	Spleen	-	-
	Skin	-	-
	Fat	-	-
Feed	BR2	+	+
	BR3	-	-

+ = positive results; - = negative results; BR2 = commercial feed mixtures containing the coccidiostat salinomycin (70 mg.kg^−1^) feed; BR3 = commercial feed mixtures without coccidiostat.

**Table 2:** Results of the mean diameters of the inhibition zones (mm ± SD) of the screening of salinomycin and its residue in matrices using the STAR method.

Sampling	Matrix	STAR				
		*B. stearothermophilus* ATCC 10149	*B. subtilis* BGA	*B. cereus* ATCC 11778	*E. coli* ATCC 11303	*K. rhizophila* ATCC 9341
A	Breast	**3.63 ± 0.28**	-	-	-	**2.34 ± 0.51**
	Thigh upper	**4.97 ± 0.59**	-	-	-	**2.09 ± 0.50**
	Thigh lower	**4.46 ± 0.45**	-	-	-	**2.13 ± 0.42**
	Heart	**7.69 ± 0.49**	-	-	-	1.56 ± 0.36
	Liver	**11.38 ± 0.43**	**2.33 ± 0.85**	-	-	**3.71 ± 0.27**
	Gizzard	**4.01 ± 0.20**	-	-	-	1.07 ± 0.42
	Kidney	**7.72 ± 1.01**	-	-	-	**1.73 ± 0.41**
	Lungs	**9.98 ± 0.95**	**2.87 ± 0.45**	-	-	1.67 ± 0.29
	Spleen	**12.22 ± 0.12**	-	-	-	**4.23 ± 0.39**
	Skin	**4.37 ± 0.18**	-	-	-	0.95 ± 0.37
	Fat	**4.54 ± 0.48**	-	-	-	1.04 ± 0.80
B	Breast	1.34 ± 0.69	-	-	-	0.77 ± 0.20
	Thigh upper	2.28 ± 0.20	-	-	-	-
	Thigh lower	2.17 ± 0.15	-	-	-	-
	Heart	2.45 ± 0.78	-	-	-	1.01 ± 0.26
	Liver	3.78 ± 0.14	-	-	-	1.13 ± 0.38
	Gizzard	1.85 ± 0.41	-	-	-	-
	Kidney	3.71 ± 0.24	-	-	-	1.14 ± 0.13
	Lungs	3.48 ± 0.27	-	-	-	1.10 ± 0.33
	Spleen	3.55 ± 0.41	-	-	-	1.54 ± 0.14
	Skin	1.43 ± 0.15	-	-	-	0.39 ± 0.14
	Fat	1.11 ± 0.23	-	-	-	-
Feed	BR2	**16.75 ± 0.31**	-	0.12 ± 0.06	-	-
	BR3	-	-	-	-	-

- = no inhibition zones; BR2 = commercial feed mixtures containing the coccidiostat salinomycin (70 mg.kg^−1^) feed; BR3 = commercial feed mixtures without coccidiostat. Bold numerals represent positive results.

**Table 3:** Determination of the mean diameters of the inhibition zones (mm ± SD) for the susceptibility of bacterial strains to STAR antibiotic control solutions.

Antibiotic	STAR				
	*B. stearothermophilus* ATCC 10149	*B. subtilis* BGA	*B. cereus* ATCC 11778	*E. coli* ATCC 11303	*K. rhizophila* ATCC 9341
SM	4.41 ± 0.58	-	-	-	-
CHTC	-	-	8.67 ± 0.25	-	-
STM	-	5.86 ± 0.36	-	-	-
TYL	-	-	-	-	6.53 ± 0.33
CF	-	-	-	8.47 ± 0.46	-

SM = sulphamethazine; CHTC = chlortetracycline; STM = streptomycin; TYL = tylosin; CF = ciprofloxacin; - = no inhibition zone.

**Table 4:** Determination of the mean diameters of the inhibition zones (mm ± SD) of sensitivity of salinomycin standards for STAR, Premi^®^Test, and Explorer 2.0 test.

Standard (µg.L^−1^)	STAR					Premi^®^ Test	Explorer 2.0 Test
	*B. stearothermophilus* ATCC 10149	*B. subtilis* BGA	*B. cereus* ATCC 11778	*E. coli* ATCC 11303	*K. rhizophila* ATCC 9341		
SAL 500	7.52 ± 0.39	-	1.37 ± 0.23	-	-	+	+
SAL 100	6.02 ± 0.41	-	-	-	-	+	+
SAL 75	2.24 ± 0.34	-	-	-	-	+	+
SAL 50	1.91 ± 0.49	-	-	-	-	+	+

- = no inhibition zones; + = positive results; SAL 500 = salinomycin standard at the concentration of 500 µg·L^−1^, SAL 100 = salinomycin standard at the concentration of 100 µg·L^−1^, SAL 75 = salinomycin standard at the concentration of 75 µg·L^−1^; SAL 50 = salinomycin standard at the concentration of 50 µg·L^−1^.

**Table 5:** Resulting concentrations of salinomycin residues determined by ELISA.

Sampling	Matrix	ELISA Salinomycin Concentration (mg.kg^−1^)
A	Breast	**0.025**
	Thigh upper	**0.032**
	Thigh lower	**0.035**
	Heart	**0.045**
	Liver	**0.241**
	Gizzard	**0.059**
	Kidney	**0.125**
	Lungs	**0.134**
	Spleen	**0.157**
	Skin	0.079
	Fat	0.082
B	Breast	0.003
	Thigh upper	0.006
	Thigh lower	0.004
	Heart	0.013
	Liver	0.076
	Gizzard	0.010
	Kidney	0.026
	Lungs	0.015
	Spleen	0.034
	Skin	0.032
	Fat	0.030

Bold numerals represent the salinomycin concentration above the MRLs.

## Data Availability

The original contributions presented in the study are included in the article, further inquiries can be directed to the corresponding author.
